# Progress of Cancer Nanotechnology as Diagnostics, Therapeutics, and Theranostics Nanomedicine: Preclinical Promise and Translational Challenges

**DOI:** 10.3390/pharmaceutics13010024

**Published:** 2020-12-24

**Authors:** Sultan Alshehri, Syed Sarim Imam, Md. Rizwanullah, Sohail Akhter, Wael Mahdi, Mohsin Kazi, Javed Ahmad

**Affiliations:** 1 Department of Pharmaceutics, College of Pharmacy, King Saud University, Riyadh 11451, Saudi Arabia; salshehri1@ksu.edu.sa (S.A.); simam@ksu.edu.sa (S.S.I.); wmahdi@ksu.edu.sa (W.M.); mkazi@ksu.edu.sa (M.K.); 2 Department of Pharmaceutical Sciences, College of Pharmacy, Almaarefa University, Riyadh 11597, Saudi Arabia; 3 Department of Pharmaceutics, School of Pharmaceutical Education and Research, Jamia Hamdard, New Delhi 110062, India; mdrizwanullah54@gmail.com or; 4 New Product Development, Global R&D, Sterile ops, TEVA Pharmaceutical Industries Ltd., Aston Ln N, Halton, Preston Brook, Runcorn WA7 3FA, UK; Sohail.Akhter@tevaruncorn.co.uk; 5 Department of Pharmaceutics, College of Pharmacy, Najran University, Najran 11001, Saudi Arabia

**Keywords:** cancer nanomedicines, theranostics, diagnostics, metallic nanoparticles, and targeted cancer chemotherapy

## Abstract

Early detection, right therapeutic intervention, and simultaneous effectiveness mapping are considered the critical factors in successful cancer therapy. Nevertheless, these factors experience the limitations of conventional cancer diagnostics and therapeutics delivery approaches. Along with providing the targeted therapeutics delivery, advances in nanomedicines have allowed the combination of therapy and diagnostics in a single system (called cancer theranostics). This paper discusses the progress in the pre-clinical and clinical development of therapeutics, diagnostics, and theranostics cancer nanomedicines. It has been well evident that compared to the overabundance of works that claimed success in pre-clinical studies, merely 15 and around 75 cancer nanomedicines are approved, and currently under clinical trials, respectively. Thus, we also brief the critical bottlenecks in the successful clinical translation of cancer nanomedicines.

## 1. Introduction

Cancer remains a significantly complex and challenging disease globally. It has been estimated that in the year 2018, the number of new cancer patients and the cancers-associated deaths reached 18.1 million and 9.6 million, respectively. Moreover, several predictive models of cancer cases are projecting 30 million deaths a year by 2030 [1]. To reduce the mortality and eventually improve patients’ survival rate, early diagnosis followed by timely and specific therapies is the key to successful cancer therapy [2]. Conventional diagnostics and therapeutic delivery lead to low bio-availability, non-targeted biodistribution, multi-drug resistance (MDR) to therapeutics, and toxicities to the healthy tissues/organs [3]. Nanomedicines advances over the years offer preferential chemotherapeutics delivery to tumors while un-exposing the healthy tissues and thus minimizing the side effects of chemotherapy and imaging agents. Various forms of organic (for example, liposomes, SLN, polymeric nanoparticles, and polymeric micelles, etc.,) and inorganic nanoparticles (NPs) [gold NPs, iron oxide NPs, carbon nanocarriers-carbon nanotubes (CNTs), and graphene, etc.,] have been synthesized that are promising in cancer-targeted delivery of therapeutics and diagnostics [4].

In principle, nanomedicines targeting cancers mainly involve three approaches: First—passive tumor targeting that exploits the tumor region characteristics termed as enhanced permeability and retention (EPR) effect; second—active targeting, in which the surface-functionalized NPs are guided to the tumors and the cancer cells by overexpressed cancer-specific ligands; and third—stimuli-responsive cancer targeting. One common ground among all these approaches is that they often require a long-biological half-life of nanoparticles upon their administration [5,6].

Recent advancements in nanomaterials synthesis and tailored made biomaterials with specific desirable physicochemical properties allow the development of “smart” nanoparticle design that can combine the diagnosis and therapy in a single carrier or system (termed as theranostics) [7]. Cancer theranostics allows faster, safer, and effective cancer therapy and remains a significant research area by cancer nanotechnology and biomedical researchers. In recent times, numerous research discussed the development and theranostic cancer nanomedicines [8,9]. This paper provides a comprehensive account of the progress made over the years in cancer nanomedicines as diagnostics, therapeutics, and theranostics. The article additionally briefs about the approved cancer nanomedicines that are currently being used in cancer therapy. Lastly, we tried to address why a large number of cancer nanomedicines (that showed a satisfactory level of safety and efficacy in preclinical studies) failed in clinical trials and the challenges in their clinical translation.

## 2. Cancer Nanotechnology: Contemporary Research in Diagnosis and Therapy

From the past few decades, continuous efforts are being made by researchers across the world for the successful development of targeted nanoprobes for the diagnosis and treatment of cancer. More recently, the advancements in nanomedicine research have fostered the fabrication of “smart” nanocarriers, which resulted in a design of nano vehicles containing both drugs and imaging agents in a single system, called nanotheranostics. It facilitates the ease in monitoring the biodistribution of drugs and flexibility for analyzing the target site accumulation of nanomedicines along with other meritorious visages like the ability to visualize and quantify triggered drug release from the nanomedicines [10]. Also, the present scenario of personalized nanomedicines-based chemotherapeutic interventions has been immensely effective in improving the patients’ life. In this section, we comprehensively update the progress made in nanomedicines research as therapeutics, diagnostics, and cancer theranostics. In recent years, different nanomaterials have gained growing attention to incorporate the radionuclides into a conventionally used nanomaterial to impart additional characteristics for application in cancer. Their ability to emit ionizing radiation has been utilized clinically not only for diagnostic but also for theranostic purposes. The efficacy of nanoparticle-mediated radionuclide therapy is associated with their ability to offer the targeted delivery of ionizing radiation for a determined period that can be utilized for curative or palliative treatment, as well as for a theranostic approach. Recently, Sakr et al. [11] and El-Ghareb et al. [12] have investigated the potential of nanoparticle-mediated radionuclide therapy in cancer for tumor imaging and theranostic purposes respectively.

**Figure 1:**
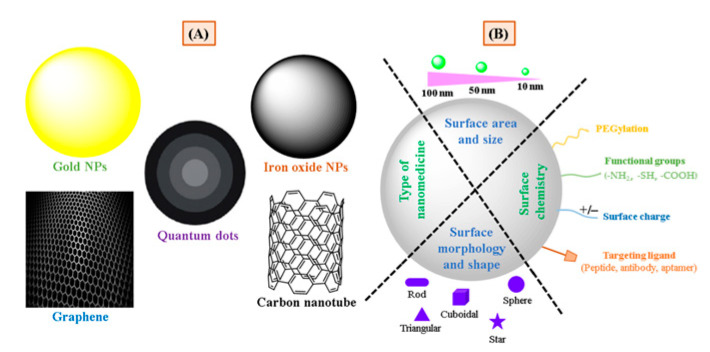
(**A**) Different types of nanomedicine utilized in cancer diagnosis and therapy; (**B**) various physicochemical characteristics of nanomedicine influencing its biopharmaceutical performance.

These nanomedicine may differ from each other based on their composition. Various nanomedicine (illustrated in Figure 1) can be categorized into those made from biodegradable materials (i.e., PLGA/PLA, chitosan, dextran, phospholipids), carbon-based materials (i.e., graphene and nanotubes), metallic NPs (i.e., which contain oxides and sulfides of metals), and semiconductor NPs (i.e., quantum dots [QDs]) [13]. Because of the unique physicochemical properties, nanomedicine is not only explored as drug-delivery nanocarriers but also as synthetic scaffolds for imaging probes used in the detection of cancers. The biopharmaceutical performance and utilization of these nanocarriers for different applications are strongly influenced by their particular chemical composition (like metallic, lipidic, polymeric, and inorganic nature) along with other structural features which include surface characteristics (like charge, and hydrophobicity), physical characteristics (like size, shape, and stiffness) and surface functionalization with specific targeting ligand and functional groups [14] (Illustrated in Figure 1). These characteristics can make nanomedicine an attractive tool in cancer management.

Indeed, from the past few decades, research in the area of cancer nanotechnology is enormously growing, while its clinical translation and approval of cancer nanomedicines for the commercial market are quite slow. Some cancer nanomedicines are approved for the commercial market and many under different phases of a clinical trial [15]. The most renowned clinically approved nanomedicines that are used in the management of different cancer, sold under the brand name of Doxil^®^/Caelyx^®^ and Abraxane^®^. Doxil^®^ is the liposomal formulation of doxorubicin, approved for the management of Kaposi’s sarcoma as well as refractory breast and ovarian cancer, while Abraxane^®^ is an albumin-based nanoparticles (NPs) of paclitaxel and approved for the management of metastatic breast cancer. As a cancer diagnostics, iron oxide NPs, Resovist^®,^ and Feridex^®^/Endorem^®^ are the approved nanodiagnostics for liver/spleen lesion imaging. Table 1 summarizes the select instances of approved nanomedicines used as chemotherapeutics and imaging agents for cancer therapy and diagnosis.

It is quite noteworthy from the list that most nanotherapeutics and diagnostics are organic-based NPs, apparently because of their biocompatibility and negligible toxicity. However, inorganic nanomedicines, such as Resovist^®^ and Feridex^®^/Endorem^®^, Aurimune^®^ (colloidal gold platform) and Auro-Lase^®^ (contains gold-coated silica NPs) have lately been approved by FDA after different phases of clinical trials [16]. Ongoing research in cancer nano-theranostics and nano-diagnostics are encouraging and have great potential to transform the cancer treatment strategy with the utmost consideration of patient compliance.

The research in cancer nanomedicines itself has been growing exponentially over the year. Generalized discussion on such a large category is beyond the focus of this article. Here, we particularly discussed the inorganic NPs/hybrid system consisting of inorganic and organic nanomaterials for its application in cancer diagnosis as well as to assess the progress of therapy as cancer theranostics. Table 2 enlists the different nanomedicine formulations investigated for the therapeutic, diagnostic, and theranostic applications in cancer and summarizes their in vitro and in vivo outcomes.

### 2.1. Nanomedicines for Cancer Therapy

The main problem with any cancer treatment is to attain the desired concentration of therapeutic agent at the tumor sites so that cancerous cells are destroyed while the damage to normal cells is minimal. Keeping this in mind, it is very important to make single agents with incredible potential to provide required input in cancer prevention, detection, and treatment. On this subject, some ligand-targeted therapeutic approaches, which include immunotoxins, radioimmuno therapeutics, and drug immunoconjugates, are in significant attention against the traditional cancer chemotherapeutics, thus providing additional tools in the depot of cancer therapy [17]. These conjugated agents show encouraging performance as compared to the traditional chemotherapy drugs, but still, limitations in their delivery remain the chief problem. Currently, it is proposed that nanotechnology, which constitutes designing and engineering of materials at nanoscale levels to form products that show new properties, would have an intense effect on disease prevention, diagnosis, and treatment. Cancer nanotechnology is an interdisciplinary research field, which bisects the areas of biology, chemistry, engineering, and medicine for the treatment and diagnosis of cancer [18]. The design of more efficient cancer therapy by engineering technology at nanoscale provides a convincing solution for the preferential removal of cancer cells without causing serious harm/toxicity to the normal cells.

#### 2.1.1. Targeted Cancer Chemotherapy

Tumor blood vessels have unique pathophysiology usually not seen in normal blood vessels. These include a quite increased quantity of proliferating endothelial cells, amplified tortuosity, lack of pericyte, as well as the aberrant basement membrane. The two primary approaches for targeting the tumor cells are *passive* and *active* targeting. These are complementary approaches, and active targeting cannot be opposed to passive targeting.

Passive Targeting: The logic behind EPR (enhanced permeability and retention)-based drug targeting is the rapid-growing leaky vascularization, and faulty lymphatic drainage, which contributes to the retention of nanoparticulate and submicron particles in tumors. Drug carriers of nano dimension that include liposomes, dendrimers, polymer-drug conjugates, polymer micelles, and inorganic NPs are widely studied in drug delivery for this particular approach in cancer chemotherapy [19]. These NPs pass through hyper-permeable blood vessels and preferentially gather in the tumor site by its EPR effect because of the required sizes (typically ranging between 1 nm and 200 nm) [20].

Active Targeting: The basis of active targeting strategy includes the interaction of ligand-stocked drug carrier with surface-exposed receptors on the target cells, which helps in their assemblage in a tumor, and also assists their intracellular accretion through receptor-mediated endocytosis [21]. Tumor cells are usually overexpressed with one or more types of specific receptors which may act as a target site for the active targeting through ligand-functionalized nanoparticles [22,23]. Thus, the tumor and endothelial cells are recognized as cellular targets for active targeting approach.

#### 2.1.2. Photodynamic Therapy

**Figure 2:**
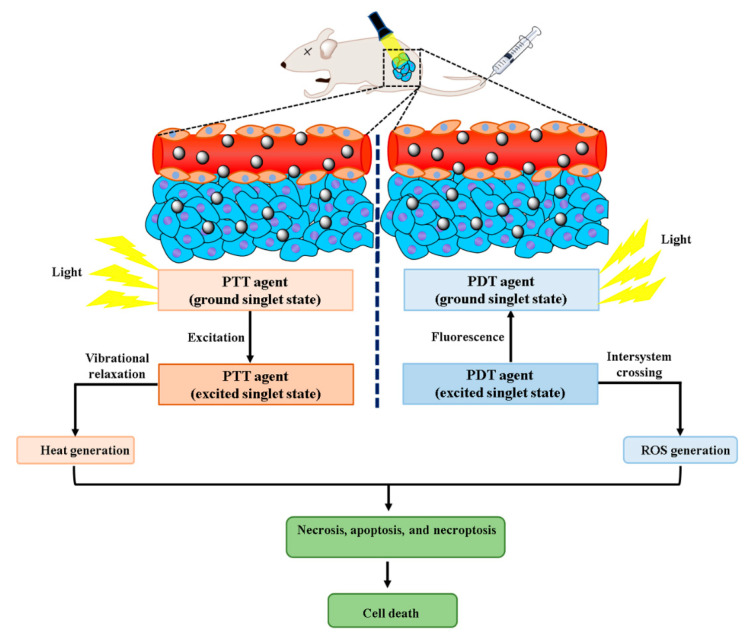
Illustration depicts the utilization of cancer nanomedicine in photothermal therapy (PTT) and photodynamic therapy (PDT) leads to cancer cell death.

Photodynamic therapy (PDT) has currently evolved as an attainable therapeutic choice in cancer chemotherapy. It exploits a photosensitizer that absorbs light of a particular wavelength and produces oxygen-based molecular species to induce a cytotoxic effect. These reactive groups harm the plasma membranes as well as subcellular organelles and lead to cell death through apoptosis, necrosis, or autophagy (illustrated in Figure 2). The photosensitizer moiety transfers their absorbed energy either to oxygen molecules to form singlet oxygen or to the neighboring molecules to generate free radicals. After that, they interact with molecular oxygen to produce hydrogen peroxide, superoxide, and hydroxyl radicals. The ability of photosensitizers to produce singlet oxygen and selectively get delivered at therapeutic concentrations at the tumor site determines the efficiency of PDT [24]. Various photosensitizers of organic nature have been utilized clinically or preclinically for PDT which includes porphyrin, chlorin or phthalocyanine derivatives, and hypericin etc. These photosensitizers are loaded in nanocarriers to improve the in vivo efficacy for nanoparticle-mediated PDT in cancer. Indeed, different nanomaterials (graphene, QDs, and titanium dioxide [TiO2] NPs etc.,) with photosensitizing properties are also exploited for PDT in recent times to overcome the limitation of various photosensitizers of hydrophobic nature [25]. The PDT is investigated for the treatment of various types of carcinoma like skin, head and neck, esophageal, stomach, pancreatic, and bladder, prostate, and lung.

Unterweger et al. developed the hypericin-containing iron oxide nanoparticles as a delivery vehicle in PDT [26]. The nanoparticles under flow cytometry study in Jurkat human T-cell leukemia cell line indicated the absence of toxicity of pure nanoparticulate system and hypericin with lack of light exposure over Jurkat T cells. However, the combined delivery of plain hypericin or NPs loaded with hypericin, followed by irradiation with light, induces concentration, and time-dependent cancer cell death because of the formation of reactive oxygen species. Moreover, Li et al. developed thiolated heparin–pheophorbide A (PhA) conjugated hybrid NPs of iron oxide and gold (Fe_3_O_4_/Au-NP) for effective monitoring of PDT [27]. The results revealed significant phototoxicity as well as strong signals of fluorescence from the A549 cancer cells treated with developed hybrid nanoparticles under light irradiation. In another investigation, Shen et al. developed a hybrid nanocomposite for tumor targeting, which consists of quantum dots-Zn-porphyrin nanocomplex. A fluorescent photosensitizer rhodamine 6G and near-infrared fluorophore (NIR775) were enclosed in folic-acid-decorated phospholipid polymers [28]. The developed system has a high payload of porphyrin, consequently resulting in exceptionally high ^1^O_2_ quantum yields. Also, in vivo study revealed the significant accumulation of developed nanoparticulate system preferentially in tumor tissue along with non-invasive fluorescence imaging for effective monitoring of PDT in mice model. Another researcher, Murakami et al. explored that the semiconducting and metallic-enriched single-walled CNTs are efficient to generate reactive oxygen species under the influence of NIR light (808 nm) irradiation [29]. The investigator reported in his findings that the semiconducting-enriched single-walled CNTs showed stronger photodynamic effects compared to the metallic-enriched single-walled CNTs.

#### 2.1.3. Photothermal Therapy

Hyperthermic treatment of tumors causes ease of cell death through protein denaturation and loosening of the cellular membrane. It is mediated by heating the tumor tissue by the application of ultrasound, microwaves, radiofrequency, and magnetic fields. The action is more specific to tumors as they are heat tolerant but it is limited because of the damage of neighboring healthy tissue [30]. Photothermal therapy (PTT) surmounts this difficulty exploiting photothermal agents which ultimately helps in precise heating of the target area and restricts the thermal damage to the tumor tissue only (illustrated in Figure 2). Photothermal agents require better light absorption and improved light-to-heat conversions for effectiveness [31]. Noble metal NPs are usually exploited as photothermal agents for in vivo treatment as a less invasive experimental technique, which assures the treatment of cancer [32]. Because of surface plasmon resonance (SPR), these possess strong absorption in the NIR regions of the electromagnetic spectrum (particularly at 650–900 nm). It conjoins two major factors: (i) light source, like lasers (spectral range of 650–900 nm) [33] for deeper penetration to the tissue as well as (ii) optical absorbing NPs, which induces photothermal ablation by effectively transforming optical irradiation to heat on picoseconds time-scale [34]. The absorption coefficients of NPs are 4–5 folds higher in magnitude compared to the photothermal dyes because of their SPR property [35]. Usually, spherical gold NPs show their maximal SPR absorption peak in the visible spectrum (approximately 520 nm).

Huang et al. validated that the Au-nanorods are efficient photothermal agents as they have a longitudinal absorption band in the NIR region because of their SPR oscillations [36]. Au-nanorods with small diameters are exploited as photothermal converters of NIR for in vivo application as they have high absorption cross-sections which pasts the absorption spectra of the tissue. The nanorods system can also be exploited as ablation components for carcinoma as they can transmit NIR light through human skin and tissue [37].

The photothermal effect in cancer cells can be increased by plasmonic silica/gold nanoshells since they generate controllable laser hyperthermia [38]. Chen et al. examined the gold nanoshell-based system for cancer targeting and PTT of HER2 over-expressing and drug-resistant ovarian cancer cells (OVCAR3) [39]. This nanocomplex system was developed for concurrent fluorescence optical imaging along with magnetic resonance imaging. It was found that the nanocomplex system irradiated with NIR laser causes the selective killing of OVCAR3 cells. Arnfield et al. reported a clinical trial observation exploiting hyperthermia induced by gold-silica nanoshell followed by exposure of NIR light to the patients suffering from oropharyngeal malignancies [40]. In another investigation, the synthesis and efficacy of palladium-gold nanostructures have been demonstrated by McGrath et al. for enhanced PTT applications [41]. The successful photothermal applications were further demonstrated in accomplishing the killing of HeLa cells under in vitro conditions and destruction of cervical cancer cells in HeLa tumor xenografts of male B9 mice by combined applications with diode laser radiations of 808 nm. Similarly, in another report, Shen et al. revealed the effect of magnetic nanoparticle clusters mediated through photothermal ablation against in vitro and in vivo models of cancer [42]. The results revealed that magnetic Fe_3_O_4_ NPs are less effective over clustered Fe_3_O_4_ NPs for accomplishing substantial enhancement in the NIR absorption. These upon irradiation of NIR at 808 nm showed induction of higher temperature for cytotoxicity against A549 cells vis-à-vis the Fe_3_O_4_ NPs. Furthermore, in vivo photothermal therapy in tumor-bearing mice model (A549) exhibited that the treatment of clustered Fe_3_O_4_ NPs has increased therapeutic efficacy as compared to the individual free Fe_3_O_4_ NPs.

### 2.2. Nanomedicine as Cancer Diagnostics

Traditional imaging systems like plain radiographs, computed tomography (CT), ultrasound, and magnetic resonance imaging (MRI) have been customarily used in both cancer screening and follow-up. Nevertheless, these modalities detect cancer after it forms a visible physical entity (~1 cm^3^ in size), by this time tumor mass comprises nearly 1 billion cancer cells [43]. Therefore, for the past one decade, anatomical imaging has been shifted to molecular imaging. Anatomical imaging locates the macroscopic/gross pathology, while molecular imaging locates cancer quite earlier at the molecular level, much before the occurrence of phenotypic changes. Molecular imaging can characterize in vivo genetic changes in oncogenesis, thus it can predict the personalized molecular therapy which would be most favorable for the patients [44]. Frequent noninvasive monitoring of cancer can also be done to check the response, progression, and transformation following therapy or recurrence.

Small molecules (nearly <2000 Daltons and 1 nm in dimension) are conventionally exploited as imaging agents in clinical practice. More commonly, 2-deoxy-2-(^18^F)fluoro-D-glucose (FDG) in positron emission tomography (PET), iodinated small molecules in CT, and chelated gadolinium in MRI are used. Research studies carried out in this domain have found its limitation as low signal intensity, nonspecific interactions, poor stability, and rapid clearance from the blood circulation [45]. NPs, however, has assured to surmount these constraints and are presently being designed as a molecular-imaging agent [46]. For instance, NPs can enhance signal intensity when optical imaging modalities are used, and thus lesser numbers of cells are imaged at larger tissue depths. It also provides stable imaging signals for a longer duration of time.

NPs show a high affinity to cancer cells since these are encrusted with a high degree of surface functionalization with ligands, thus permitting multiple cellular bonds with the target tissues. This increases their association constant by 4–5 folds of magnitude [47]. This increases the signal-to-noise ratio, permitting NPs to gather at the tumor site and the cancerous tissue to be highlighted more as compared to the adjacent normal tissue, thus proving it to be highly beneficial. The majority of NPs-imaging agents are large (>10 nm) and are therefore not removed by typical renal circulation [48]. Therefore, they have circulation times longer as compared to the small molecules (i.e., days vs. minutes). It is quite useful in repeated imaging as no further NPs administration is required. Fascinatingly, it has been found in studies that smaller NPs show more uniform tissue biodistribution. Studies have also shown that non-spherical NPs (like nanotubes, nanodisks, nanoworms, etc.,) are comparatively proved to be the more efficient-target delivery agents than spherical nanoparticles [49,50]. However, this needs to be counterpoised against the significantly augmented toxicity caused by the non-spherical NPs [51,52]. Since single molecular alteration rarely results in cancer, therefore concurrent detection of multiple molecular targets is upregulated in the course of the oncogenesis process (i.e., a process also called multiplexing) for increasing the specificity of cancer cell detection. It can also be achieved by labeling various NPs and then administering all the NPs at a time. The signals discovered from these NPs bound to the cancer cells can be used to decode a molecular profile for cancer to be identified. Consecutively, a molecularly targeted therapy can be conceptualized and meted out to the patient. A novel approach is if the cancer molecular profile is already known to label a single NP with multiple different ligands, each directed toward a specific molecular target, known to be upregulated by the tumor being explored. Since tumors will have more number of targets as compared to the surrounding tissues, it will attach more NPs and generate stronger signals. As a result, NPs can be designed as multimodal approaches that can be imaged by two or more types of imaging modalities (like, MRI and fluorescence). Some research groups have investigated different methods to inject individual subcomponents or building blocks of NPs so that the delivery efficiency of the NPs imaging agents can be increased. These subcomponents can conglomerate to form supramolecular NPs because of the presence of triggering agents like pH adjustment, reduction, or enzymatic cleavage. These supramolecular NPs probes can be used for imaging agents [53,54]. This strategy is highly advantageous as the individual subcomponents will be tiny, and it will maximize the accumulation at the target site because of better admittance to the tumor cells. For instance, gadolinium-containing monomers, which gather in cells through thiol-sensitive reduction of 1,2-aminothiol and 2-cyanobenzothiazole ligands with specific binding ability to furin and caspase-3 overexpressed on the tumor cells [55].

#### 2.2.1. Gold NPs

Gold NPs (AuNPs) have biocompatibility among numerous useful attributes, which make them highly attractive contrast agents for cancer chemotherapy. Akhter et al. through extensive reviews have categorically exemplified the diagnostic, therapeutics, and theranostics application in cancer therapy [56]. AuNPs are used in CT imaging and as adjuvants in radiotherapy, as these attenuate X-ray radiations. Nucleic acids are broadly detected by fluorescent organic dyes. Nevertheless, these dyes can be detected by fewer techniques, as these undergo photobleaching. Alternatively, photobleaching is not a problem for AuNPs, and also their absorption and scattering cross-sections are better than those of conventional dyes. The exclusive optical characteristics of AuNPs permit the detection of zeptomolar concentrations of nucleic acids, thus provides greater sensitivity of five orders of magnitude as compared to the fluorescence-based techniques [57]. AuNPs are a novel marker for molecular diagnostic and are widely used for precise determination of DNA and also for marking single strands of the target DNA [58]. These AuNPs marked single-stranded DNA fragments are restrained in a narrow electrode gap of sensor devices. As a result of hybridization, double-stranded DNA fragments are formed, which contribute to the conductivity change across the gap junctions and hybridization event is sensed.

Spherical gold particles perused along with DNA form the basis of easy-to-read test for the occurrence of genetic sequences. Complementary DNA of half of such sequence gets enclosed to one set of the particle in solution, and complementary DNA of the other half is enclosed to another set of particles. If the sample contains the desired sequence, then it will get attached to DNA on both sets of particles, thus confines the particle in the web and causes the solution to change the color [59]. Moreover, a change in the size, shape, and composition of AuNPs permits the modification of their optical characteristics, unlike conventional dyes, and allows their use for concurrent detection of multiple targets. Different techniques like colorimetric, scanometric, and electrical detection techniques are used to detect AuNPs. This makes their use appealing in a variety of biomedical applications including molecular diagnostics [60]. Various forms of gold nanomaterials recently explored include gold nanocage, gold nanoshell, and gold nanorods, etc. Numerous research studies have been conducted on the possibility of AuNPs as imaging agents.

Zhou et al. developed the PEGylated polyethyleneimine-entrapped AuNPs conjugated with folic acid for cancer targeting and CT imaging [61]. In vitro flow cytometry and confocal microscopy imaging studies revealed that developed diagnostic systems can target the cancer cells (KB-HFAR and KB-LFAR) overexpressed with folate receptors. The developed diagnostic system in in vitro (KB-HFAR and KB-LFAR cell line) and in vivo model (xenografted tumor model) demonstrated remarkable improvement in CT contrast in comparison to the non-targeted AuNPs. In another investigation, Liu et al. fabricated lactobionic acid conjugated dendrimer-entrapped AuNPs for targeted CT imaging against liver cancer [62]. In vitro flow cytometry analysis revealed that the developed diagnostic systems were specifically cellular uptake by the cancer cells (HepG2 and L929 cells) having overexpression of asialoglycoprotein receptors. The developed diagnostic system was found to exhibit more significant improvement in CT contrast characteristics at the same concentrations (200 nM or above) compared to a non-targeted diagnostic system in both the in vitro (HepG2 cells) and in vivo (xenografted tumor model) investigation. It is more likely because of the lactobionic acid-mediated-specific binding and cellular uptake of the AuNPs. Furthermore, the developed AuNPs-based diagnostic system exhibited significantly higher X-ray attenuation characteristics over the clinically approved iodine-based CT contrast agents. Similarly, Chen et al. developed and characterized folic acid-grafted multifunctional dendrimer-entrapped AuNPs having gadolinium for tumor-targeted multimodal CT and MR imaging [63]. The developed system demonstrated high X-ray attenuation intensity and rational *r*_1_ relaxivity because of the co-existence of AuNPs and gadolinium ions within the single system. These characteristics of the NPs help them to be used as dual-mode nanoprobes for cancer-targeted CT and MR imaging in in vitro (kB-HFAR cells and KB-LFAR cells) and in vivo (xenograft tumor-bearing BALB/c nude mice) conditions.

#### 2.2.2. Iron Oxide NPs

Even though many studies are presently undertaken preclinically to produce the new NPs agents, superparamagnetic iron oxide nanoparticles (SPIONs) have already been used as imaging agents for many diseases associated with hepatic, cardiovascular, and lymphatic systems in clinical practice. Iron oxide (magnetite, Fe_3_O_4_; maghemite, Fe_2_O_3_) NPs developed into the superparamagnetic NPs at room temperature possess their core dimension of 20 nm or less [64]. This enhances the MRI contrast property, as it permits the vulnerability action even at µM concentrations which modify the T_2_ and T_2_* relaxation times of water protons [65].

Moreover, the SPIONs are appealing as they possess more magnetic susceptibility than conventional MR contrast agents (like gadolinium). This type of particulate system has quick hepatic uptake upon intravenous administration and can be effectively used for the identification of liver cancer [66]. Ultrasmall SPIONs of 5–10 nm have prevalent tissue distribution property, thus permitting their uptake in lymph nodes and bone marrow [67]. Clinically, it has been exploited in humans to distinguish the lymph node status in patients suffering from cancer of the mammary gland [68], lung [69], prostate [70], endometrium, and cervix [71]. These are exploited in combination with high-resolution MRI techniques to identify the small and other undetectable lymph node metastases [72].

Hu et al. developed the RGD (arginine-glycine-aspartic acid) peptide-targeted iron oxide NPs for MR imaging of tumor cells [73]. The developed NPs were found to be colloidally stable, hemocompatible, as well as cytocompatible. Flow cytometry and confocal microscopy confirm the selective targeting potential of NPs to αvβ3 integrin overexpressed cancer cells. Moreover, the developed multifunctional NPs displayed extremely high r2 relaxivity during MR imaging in both in vitro (U87MG cells) and in vivo (xenograft tumor-bearing BALB/c nude mice) conditions. Similarly, Li et al. reported a polyethyleneimine-mediated approach for synthesizing the hyaluronic acid-targeted iron oxide NPs for the MR imaging of tumor cells under in vitro and in vivo conditions [74]. The confocal microscopy and flow cytometric analysis revealed that the developed diagnostic system specifically uptaken by the cancer cells overexpressed with the CD44 receptors (Hela cells). Furthermore, the developed multifunctional diagnostic NPs exhibited relatively high r_2_ relaxivity and excellent enhancement of contrast in T_2_-weighted MR imaging of cancer cells in a xenografted tumor-bearing mice model through hyaluronic acid-mediated active targeting approach. In another investigation, Dai et al. designed and developed PEG-coated SPIONs by a facile one-pot approach [75]. The developed PEG-SPIONs were found to be colloidally stable at a wide pH range and remain stable at high ionic-strength. PEG-SPIONs further showed excellent superparamagnetic behavior. The MRI characteristics of PEG-coated SPIONs were evaluated under in vitro (NIH-3T3 cells) and in vivo (in Kunming mice) conditions. The developed SPIONs showed dual contrast both in T_1_ and T_2_-weighted imaging with longitudinal and transverse relaxivity. It was found that T_2_-weighted MRI exhibited significantly higher enhancement in the liver and spleen compared to T_1_-weighted MRI. In vivo biodistribution profile in Kunming mice revealed that the developed PEG-SPIONs show gradual clearance via hepatobiliary processing. Moreover, PEG-SPIONs did not show any toxicity and were safe for MRI.

#### 2.2.3. Quantum Dots

Currently, semiconductor NPs consist of II-VI or III-V group metals, known as QDs. It is widely used as imaging and labeling agents in cancer therapy [76]. Indium arsenide, cadmium telluride, and cadmium selenide are the frequently used compounds for this purpose. QDs are useful for their multicolor imaging characteristics with a single excitation source because of their broad absorption and narrow emission property. QDs are good contenders for fluorescent tagging for their in vivo molecular and cellular imaging, as they have high fluorescence quantum yield, photobleaching resistance, and exceptional physical, chemical, and optical properties [77].

Tang et al. developed the tumor-avid cyclic pentapeptide-labeled NIR emitting silver sulfide-QDs for integrin-targeted cancer imaging [78]. It was found that the selective integrin-mediated internalization was observed in cancer cells treated with the peptide-labeled QDs only. Moreover, non-targeted NPs exhibited negatively charged fluorescent dye molecules, which typically do not internalize in the cancer cells. The biodistribution profiles of QDs after intravenous administration in in vivo (Balb/c mice) conditions revealed a remarkably high tumor-to-liver uptake ratio. It indicates that the tiny dimension of QDs avoids opsonization, resulting in increased hepatic uptake. Similarly, Li et al. successfully developed antibody (BRCAA1 and Her2)-conjugated amphiphilic polymeric engineered QDs of CdSe/ZnS for imaging against gastric carcinoma [79]. These results demonstrated that the developed system exhibits strong photoluminescence and good biocompatibility properties along with targeted imaging of gastric cancer (MGC803) cells. In another investigation, Foda et al. developed the biocompatible and highly luminescent QDs of CuInS_2_/ZnS embedded silica beads for imaging of cancer cells [80]. The developed diagnostic system exhibited dominant NIR band-edge luminescence at 650−720 nm with a quantum yield between 30 and 50 percent. It exhibited significant photoluminescence and colloidal stability in aqueous media.

#### 2.2.4. Gadolinium-Incorporated NPs

Gadolinium-containing MRI contrast agents can function at approximately 100 times less concentration than iodine atoms required for CT imaging. The sensitivity of imaging can be improved by targeting a single site [81,82]. Generally, more than 25% of the cases are treated by a newer contrast material which is known as gado-nanotubes (contains the metal gadolinium) [83]. In these NPs, the metal atoms are enclosed within a hollow core of CNTs, protecting the patients from the metal toxicity. The gadolinium atoms accumulate to form clusters inside the tubes, where a novel group of MRI contrast agents was found to be 40–90 times more responsive compared to any gadolinium-based contrast agent presently used in clinical practice.

Xu et al. targeted ovarian cancer in vivo by synthesizing a G2 PAMAM dendrimer combined with Gd(III)-1B4M-DTPA. Rhodamine green was used to fluorescently mark the dendrimer for MRI and optical fluorescence imaging. This nanodiagnostics indicates efficient targeting to the tumor tissue and releases adequate amounts of chelated Gd(III) and fluorophore to the tumor-producing visible changes in the tumor tissue by MRI and fluorescence imaging, when it was given to mice having ovarian tumor xenografts [84]. Similarly, Kim et al. developed the pullulan-based gadolinium-chelated NPs for delivery to human mesenchymal stem cells by the photochemical-internalization technique for the diagnosis of cancer [85]. The gadolinium entrapped human mesenchymal stem cells showed early detection of the tumor (~3 mm^3^) within 2 h after administration of NPs within the tumor (CT26 cells)-bearing mice model through MRI and optical imaging. In another investigation, Mi et al. developed PEGylated calcium phosphate NPs incorporated with diethylenetriaminepentaacetic acid gadolinium (III) and exhibited enhanced MRI diagnosis characteristics for identifying the solid tumors [86]. The developed NPs in C-26 tumor-bearing BALB/c nude mice exhibited >40% enhancement in the signal intensity for the detection of tumors after 4 h of intravenous administration. Moreover, NPs revealed a >6-fold increase in relaxivity compared to the free form.

#### 2.2.5. Carbon Nanotubes and Graphenes

Carbon-based nanomaterials, namely CNTs and graphenes have been studied extensively at the pre-clinical level in cancer therapy R&D and showed promising results as cancer diagnostics. We are going to discuss here the diagnostic and theranostic applications of CNTs and graphenes in cancer. In this section, we only discuss their diagnostics potential explored in cancer therapy. The theranostics activity of these carbon-based NPs is discussed later in a separate section. CNTs are the nanodevice used for the detection of cancer biomarkers [87]. These are carbon cylinders having benzene rings arranged in a regular fashion [88]. It has powerful optical absorbance in the high-near infrared region of the electromagnetic spectrum (i.e., ranging between 700 and 1100 nm), thus highly useful as the photoacoustic and optical imaging agents [89]. Graphenes and their functionalized derivatives are also used as fluorescent markers for intracellular imaging studies. Magnetic NPs containing graphene and their derivatives are also used for MRI applications in cancer diagnosis [90]. Recently, ultra-small sized graphene-quantum dots have been developed for utilizing their fluorescent emission properties for biomedical imaging [91].

Hou et al. developed the hyaluronic acid (HA)-functionalized SWNTs to deliver the contrast agent, GdCl_3_, for tumor targeting and MRI purpose [92]. Remarkably superior results were observed for HA-SWNTs exhibiting significantly enhanced cellular uptake as compared to the plain SWNTs in MCF-7 mice melanoma cell line owing to the presence of HA. Moreover, GdCl_3_-bearing HA-SWNTs showed significantly higher circulation time for MRI. In vivo imaging in S180 cells, tumor-bearing male BALB/c mice revealed that the developed system exhibited the highest tumor-targeting efficiency and T1-relaxivity enhancement. In another investigation, Rubio et al. evaluated the intrinsic nonlinear photoluminescence characteristics of chemically functionalized MWNTs for cancer cells (A549) imaging under in vitro and in vivo conditions [93]. Moreover, solid tumors were identified using multiphoton photoluminescence and fluorescence imaging. The results showed improved T2 relaxivities for the hybrid material (186 m_M_^‒1^ s^‒1^) compared to the pure magnetic NPs (92 m_M_^‒1^ s^‒1^) because of the capacity of MWNTs to “carry” more NPs as clusters. It was found that intravenous administration of the composite system in in vivo liver cancer model in mice resulted in a remarkable increase in tumor to liver contrast ratio (277%) in T2 weighted MRI.

**Figure 3:**
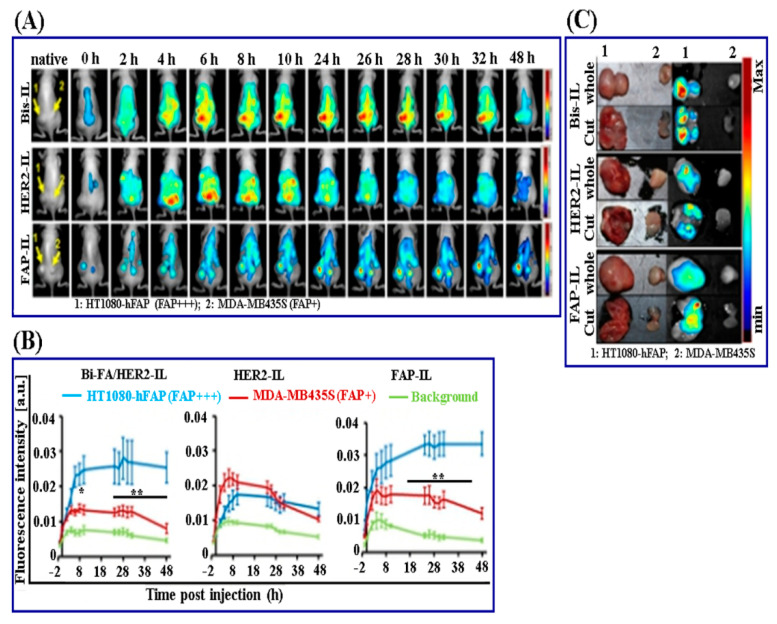
Illustration depicts in vivo evaluation of near-infrared fluorescent dye-loaded liposomal nanomedicine as cancer diagnostics for the image-guided nuclear delivery of the encapsulated dye (**A**) in vivo imaging of delivery of NIR-fluorescence dye (DY676-COOH) in mice bearing FAP-expressing fibrosarcoma HT1080-hFAP [1] and FAP-expressing human melanoma MDA-MB435S [2] after i.v. injection for 0–48 h. (**B**) Semiquantitative analysis of fluorescence intensities of a region of interest on respective tumors or background (thigh region) at a given time point. (**C**) Photograph and NIR-fluorescence images of tumor tissue excised after the 48 h of liposomal nanomedicine (monospecific and bispecific) administration indicating intense liposomal fluorescence in case of mice bearing FAP-expressing fibrosarcoma HT1080-hFAP. Reproduced from [94], MDPI, 2020. Note: Three groups of tumor-bearing mice were treated with two types of monospecific liposome (HER2-IL and FAP-IL) and one Bispecific liposome (Bi-FAP/HER2-IL). * significant compared to control, ** highly significant compared to control.

More recently, researchers are also exploring the potential of different forms of organic nanomedicine such as liposomes, SLN, polymeric nanoparticles, and polymeric micelles, etc., for cancer-targeted delivery of diagnostics. Tansi et al. developed near-infrared fluorescent dye (DY676-COOH) loaded liposomal nanomedicine as cancer diagnostics for the image-guided nuclear delivery of the encapsulated dye [94]. This investigation involves the design and evaluation of two types of monospecific liposomes (HER2-IL and FAP-IL) and one bispecific liposome (Bi-FAP/HER2-IL) subjected to physicochemical characterization, in vitro cellular uptake validations, and in vivo fluorescence imaging of xenograft models in mice. The in vivo image-guided delivery of NIR-fluorescence dye in mice bearing FAP-expressing fibrosarcoma HT1080-hFAP and FAP-expressing human melanoma MDA-MB435S was carried out. It was found that tumor tissue excised after the 48 h of liposomal nanomedicine administration indicates intense liposomal fluorescence in the case of mice bearing FAP-expressing fibrosarcoma HT1080-hFAP compared to the FAP-expressing human melanoma MDA-MB435S (Illustrated in Figure 3).

### 2.3. Nanomedicine as Cancer Theranostics

**Figure 4:**
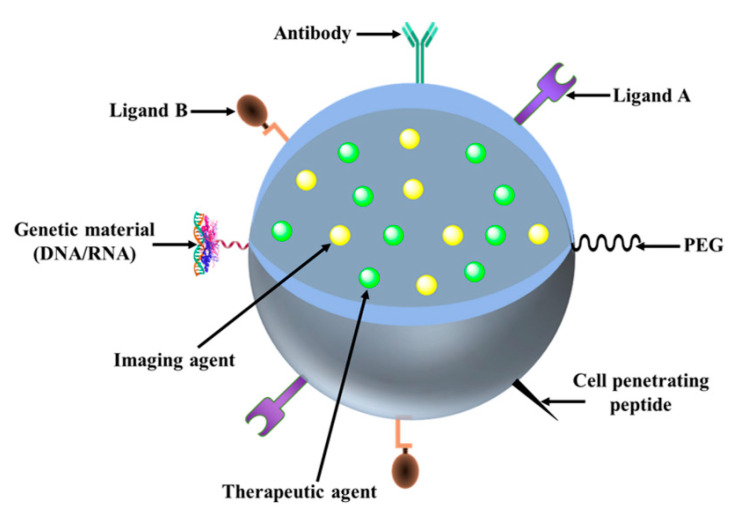
Illustration depicts diagnostic/imaging agent and therapeutic agent consist in a single NPs system as cancer theranostics.

Theranostics combines the merits of the diagnostic and therapeutic ability of an agent in a single system such as NPs (illustrated in Figure 4). The concept of theranostic reflects upon the designing of NPs in a way that it can simultaneously diagnose, treat, and monitor the therapy response in a single integrated system [95]. Such multifaceted nanoparticles are anticipated to raise drug development to new heights with minimized risks and costs. The emerging polymerization and emulsifying techniques allow nanoparticles to be formulated with hydrophilic and hydrophobic surfaces, which in turn enable their payload with various active materials (i.e., a contrasting agent of hydrophilic nature while a therapeutic agent of hydrophobic nature and vice versa). The prospect of nanomedicine relies upon multiple functioning nanoplatforms that integrate the therapeutic aspect with multimodal imaging ability. Such synchronization of detection capability with therapeutic intrusions is essential to overcome the hurdles of cancer heterogeneity as well as an adaptation [96]. The paramount objective of nanomedicine is to enable nanoparticle-based agents to deliver payload (radioisotopes, drugs, genes, etc.,) efficiently and specifically avoiding systemic toxicity to measure non-invasively delivered therapeutic efficacy over time with accuracy. In a study reported by Shim et al. the designed RNA-encapsulated polyplexes were attached covalently with gold NPs via acid-labile linkage to investigate their theranostic utility [97].

#### 2.3.1. SPIONs

In light of reported literature, primarily two forms of iron oxide nanoparticle (superparamagnetic iron oxide [SPIONs] and ultra-small superparamagnetic iron oxide [USPIONs]) can be applied as imaging agents [98]. The foremost benefits of SPIONs include biocompatibility and biodegradability characteristics of iron, as it can be reutilized through a normal pathway of iron metabolism [99]. At present, peptide conjugated SPIONs are effectively targeted on transferrin and pancreatic receptors overexpressed cells. Moreover, synaptotagmin conjugated SPIONs facilitated the detection of the cells suffering from chemotherapeutic apoptosis [100].

Medarova et al. confirmed the NPs-mediated transfection of siRNA and its concomitant localization imaging in the tumor cell through MRI and NIR optical imaging [101]. The developed theranostic system was SPIONs as a magnetic NPs (for MRI), labeled with Cy5.5 dye (for NIR imaging) and covalently linked to siRNA molecule for dual-purpose probes for siRNA delivery and simultaneous in vivo imaging of its accumulation in tumor tissue through MRI and NIR imaging. It was conjugated with myristoylated polyarginine peptides (MPAP) which act as a membrane translocation module for intracellular delivery of siRNA. The developed theranostic system is helpful to attain substantial silencing in tumors of mice bearing subcutaneous LS174T human colorectal adenocarcinoma. In another investigation, Maeng et al. developed a doxorubicin-loaded hybrid system composed of inorganic (SPIONs) and organic (poly (ethylene oxide)-trimellitic anhydride chloride-folate) nanomaterials for cancer-targeted delivery and imaging against liver cancer. Certainly, this folate-coated hybrid NPs demonstrated improved anticancer activity (2-fold and a 4-fold decrease in tumor volume) by targeting folate receptor-overexpressed tumors in comparison to free doxorubicin and DOXIL^®^ treated group in rat and rabbit models [102]. The developed theranostic system exhibited higher MRI sensitivity comparable to Resovist^®^ (conventional MRI contrast agent), even in its lower iron content. The designed system has significant potential for treating liver cancer and acts as a promising theranostic candidate to monitor the progress of cancer exploiting the MRI technique. Different researchers have investigated the utility of epirubicin-5TR1 aptamer-SPIONs as a tertiary complex system for the treatment and imaging of murine colon cancer (C26) cells [103]. The epirubicin or epirubicin-Aptamer-SPIONs as the tertiary complex system was shown to treat C26 and CHO-K1 cells in MTT assay. Study findings revealed that the developed system was efficiently internalized into C26 cells, but not into the CHO-K1 cells as confirmed through flow cytometric analysis. Further, MRI demonstrated high internalization of the developed system within the tumor. Consequently, epirubicin-Aptamer-SPIONs as a tertiary complex system is presented as an efficient targeted delivery system of epirubicin to C26 cancer cells. Furthermore, the developed system could successfully detect tumors as well as impede tumor growth under in vivo environment. Nafiujjaman et al. developed multifunctional pheoA (photosensitizer) conjugated heparin-iron oxide nanoparticles (PheoA–Hep–Fe_3_O_4_ NPs) for investigating the feasibility for effective PDT applications and dual-mode fluorescence imaging of cancer cells using MRI [104]. Markedly high fluorescent intensity was observed from the PheoA–Hep–Fe3O4 nanoparticles in KB cells, indicating a high degree of internalization potential of the NPs as compared to the free PheoA. Moreover, the MR imaging capability of PheoA–Hep–Fe3O4 NPs showed an increase in the signal intensity with an increase in the concentration of Fe, leading to high signal intensity for the detection of MR images. On the other hand, PheoA–Hep–Fe3O4 nanoparticles showed higher intensity than Fe_3_O_4_ nanoparticles ostensibly owing to high water stability during the relaxation process. The application of PDT also showed dose and light-dependent cytotoxicity after 24 h exposure of KB cells to PheoA–Hep–Fe3O4 nanoparticles, followed by laser irradiation for the purpose. In another instance, the efficacy of folic acid-targeted, photosensitizer-loaded iron oxide nanocomposites have been studied in vivo for their T2-weighted MRI property and the visual ability for PDT in imaging the tumor (MCF-7 cancer cells)-bearing nude mice model [105]. Further, Yu et al. demonstrated the ability of magnetic NPs targeted photothermal therapy in cancer for MRI and PAT imaging by the integration of MoS2 flakes and NPs of Fe_3_O_4_. Among these, NIR light was converted into heat by MoS2 while NPs of Fe_3_O_4_ served as target moiety for the external magnetic field at the tumor site [106]. Zhou et al. developed the PEGylated iron/iron oxide core/shell NPs to impart triple functional characteristics in one entity including drug targeting, photothermal therapy, and MRI of cancer cells [107]. The results revealed that the developed system exhibited comparable photothermal conversion efficiency and significantly higher photothermal stability as compared to the Au nanorods. Overall, the intensity of the MRI signal was found to be improved by ~3 folds and temperature increased by ~2-folds as compared to the lack of magnetic targeting in a tumor (HeLa)-bearing nude mice.

#### 2.3.2. Gold Nano-Theranostic

The current scenario is flooded with various inorganic NPs, among which gold NPs hold superiority as a promising carrier for their outstanding optical and photoelectric characteristics, high stability, inert nature and lack of toxicity issue, ease of preparation, the possibility of bio-conjugation as well as bio-modification with disulfides, thiol, and amino groups. The conjugation with thiolated-PEG further enhances their dispersibility [108]. The most dynamic areas of AuNPs applications include diagnosis, where they can be used as contrast agents and in photothermal cancer therapy. The exposure to light causes interaction with the oscillating electric field component of light with the electrons (free conduction-band) present at the surface of the AuNPs. It initiates their collective dipolar oscillation known as a surface plasmon. When these surface plasmons gain a frequency identical to that of the excitation light, the phenomenon is referred to as surface plasmon resonance (SPR). This SPR is attributed to the irreversible cellular damage in a localized area due to thermal effect and imaging via light irradiation of suitable wavelength on the accumulation of AuNPs at the target site [109]. These aspects mark tremendous theranostic prospects for AuNPs as nanometric systems for treatment in deep-seated cancerous tissues. PEG-coated AuNPs (approximately 30 nm in dimension) were applied for CT contrast agents and demonstrated an enhancement in the contrast imaging potential in in vivo system. It ultimately causes a reduction in the required radiation dose. In addition to this, the PEG-coated AuNPs also overcome the general limitations of conventional contrast agents (like iodine-based compounds) which include short imaging time because of fast renal clearance, vascular permeation, and kidney toxicity [110].

Zhao et al. developed gold nanochains (AuNCs) as a potential theranostic system having surface-enhanced Raman scattering (SERS) and utilized it for the multiplex detection as well as PDT of cancer [111]. AuNCs demonstrated high absorption in the NIR region and effective SERS characteristics. It showed >99% cellular uptake and demonstrated significant phototoxicity in HeLa cells even at a low concentration of photosensitizers in comparison to free photosensitizer concentration under laser irradiation. In another investigation, Nair et al. designed folic acid conjugated gold quantum cluster capped with lipoic acid for the fluorescence imaging-guided PDT in cancer [112]. It revealed that photosensitizers in the developed system generate significantly high singlet oxygen yield compared to the free photosensitizer (protoporphyrin IX). The better localization of the developed theranostic system in tumor tissue and high singlet oxygen yield facilitated targeted cell death with a sufficiently low dose of laser irradiation. Indeed, the NIR emission ability of developed nanotheranostics facilitates real-time tracking of the progress of PDT. The in vivo investigation in xenograft of Albino Swiss mice bearing C6 cell line demonstrated that the designed nanocluster system is a very useful tool for the monitoring and destruction of the tumor cells. In another investigation, Srivatsan et al. has designed and developed PEG-coated gold nanocages for the fluorescence and photoacoustic dual-modal image-guided PDT in cancer [113]. In vitro fluorescence imaging study revealed that developed nanocage system showed remarkably higher cellular uptake in C26 colon cancer cells compared to free photosensitizers [3-devinyl-3-(1′-hexyloxyethyl)pyropheophorbide]. Also, the tumor boundary and tumor-feeding vasculature are visualized from photoacoustic imaging exploiting developed nanotheranostics. In vitro efficacy of developed nanotheranostics in C26 cell line revealed an approximately two-fold reduction in IC_50_ value compared to free photosensitizers. In vivo efficacy of developed nanotheranostics in tumor-bearing BALB/c mice exhibited 73% enhancement of ^1^O_2_ signal in comparison to the same amount of free photosensitizers upon light irradiation at 665 nm. The relatively high potency of the developed nanotheranostics consisting of photosensitizers is due to the enhancement in the generation of ^1^O_2_ and/or improved tumor cellular uptake in comparison to the free photosensitizers.

#### 2.3.3. Quantum Dots

The broad excitation spectra with narrow and tunable emission spectra, larger molar extinction coefficients, exceptionally high photochemical stability of QDs compared to the organic dyes make them very useful tools for the theranostic application in cancer. Various studies indicate that QDs are widely exploited for theranostic applications in cancer.

Xia et al. designed and developed multifunctional theranostic NPs by incorporating gold nanorods and QDs of CdSe/ZnS into silica and folic acid being a targeting ligand for the dual-modality CT and fluorescence imaging along with PTT in cancer [114]. The MTT assay revealed low cytotoxicity of these NPs and confocal fluorescence images illustrated that the folate receptors-overexpressed cancer cells (HeLa cells) selectively targeted rather than folate receptors-deficient A549 cells. It was also found that these NPs demonstrated strong X-ray and fluorescence attenuation for the CT imaging and fluorescence imaging respectively in cancer cells. It also exhibited an improved PTT effect against cancer cells because of the high absorption coefficient of gold nanorods in the NIR region as well as a better rate of heat generation. In another investigation, Yong et al. designed and developed QDs of tungsten sulfide multifunctional nanotheranostic system for the dual-modal imaging-guided synergistic photothermal and radiotherapy against cancer disease [115]. The developed nanotheranostic system was found to remarkably enhance the signals in CT and photoacoustic imaging as well as was significantly effective against in vitro cancer cell lines (HeLa and HepG2 cells). Also, a nanotheranostic system was developed which was helpful to accurately position and eradicate the targeted tumor in BEL-7402-bearing BALB/c nude mice after the administration of an intravenous injection. Indeed, histopathology, blood hematology, and biochemical analysis of the developed nanotheranostic system revealed no significant toxicity in in vitro and in vivo investigation.

#### 2.3.4. Carbon Nanotubes, Carbon Dots, and Graphene Theranostic

Nano-carbon allotropes (nano-diamonds, fullerenes, CNTs, carbon nanoparticles, and graphenes) belong to a class of nanostructures that has been taken into notice in the past two decades for biomedical applications [116]. The inherent optical characteristics like fluorescence and photoacoustic emission make them a valuable contrast agent in optical imaging as well as biosensing [117]. Despite the toxicity of CNTs, these are used in biomedical applications after PEGylation by covalent or non-covalent methods to reduce their toxicity [118]. Although, internalization mechanism of the CNTs into the cells is not well understood, yet they are assumed to enter into the cells independent of the availability of surface functional groups and cell type. It is also used in antitumor immunotherapy, where antigen-presenting carriers can enhance the humoral immune response against tumor-bearing patients [119]. The report suggested that in cell culture as well as xenograft mice models, cationic CNTs are useful as siRNA molecular transporters to stop the expression of the specific gene [120].

A nano-graphene oxide (nGO) was conjugated with anti-HER2 antibody (trastuzumab) and radiolabeled with indium^111^ as well as a metal-ion chelator to improve the imaging action against HER2-overexpressed human breast cancer cells in comparison to the radiolabeled trastuzumab without nGO [121]. Also, nGO was utilized for the ^66^Ga-labeling and demonstrated significantly improved targeting potential against tumor in 4T_1_ murine breast tumor-bearing mice [122]. It is also exploited for PET-guided imaging and photothermal effect because of graphene oxide. The developed system may exploit for molecular targeted drug delivery and PTT to improve the therapeutic efficacy as a cancer theranostics. In another study, an enzyme-activating graphene oxide-photosensitizer complex was conjugated to hyaluronic acid and demonstrated better imaging potential under NIR light and significantly improved PDT effect against human A549 lung cancer cells having overexpression of lysosomal HAdase [123]. The developed combination system induces a mild increase in temperature using laser radiation of 810 nm and causes 33% of tumor cell death. This provided a proof-of-concept for the enzyme-specific graphene–photosensitizer combination system acting as an efficient tool for theranostic application in cancer.

A multi-walled CNTs coated with manganese oxide and polyethylene glycol was developed by Wang et al. as a cancer theranostics against metastatic lymph nodes [124]. The developed system exhibited significant outcomes in in vitro cytotoxicity assay against A549 cancer cells and very high photothermal conversion efficiency. The developed theranostic system in in vivo investigation in metastases (A549)-bearing nude mice demonstrated strong dual-modality (MRI and dark dye imaging) lymphatic tracking capability as well as remarkable efficiency for the tumor ablation. Also, Zhang et al. designed and developed PEGylated graphene oxide-BaGdF_5_ nanocomposites for multi-modal imaging and PTT in cancer [125]. The enhanced NIR absorbance, better photothermal stability, and improved passive targeting efficiency of developed nanocomposites resulted in effective photothermal ablation of tumor in HeLa cell-bearing nude mice after the administration of intravenous injection along with 808 nm laser irradiation. In another investigation, Nurunnabi et al. demonstrated the potential of carboxylated photoluminescent graphene nanodots (cGdots) for simultaneous imaging and PDT/PTT application in cancer [126]. The developed cGdots exhibited significant thermal ablation at a wavelength of 670 nm and generated singlet oxygen similar to pheophorbide-A to exert PDT action against MDA-MB-231 cancer cells. The findings of in vitro cell cytotoxicity assay revealed the killing of 70% MDA-MB-231 cancer cells under the combined action of photodynamic and photothermal effects. The developed cGdots in in vivo investigation in MDA-MB-231 xenograft tumor-bearing mice have demonstrated a 70% reduction in tumor volume in 21 days after the intravenous injection and tumor tissue could be easily visualized in mice as optical imaging-guided therapy.

Fluorescence imaging via carbon dots (CDs) has found multifunctional applications in cancer imaging, drug delivery as well as tracking response of the therapy. This latest generation of carbon nanomaterials with substantial surface area, high quantum yield, multicolor wavelength emission, improved water stability, and high drug loading efficiency, have gained a growing interest as promising cancer theranostics compared to QDs [127]. Zhang F et al. investigated the cancer theranostic potential of berberine-loaded CDs against the xenograft tumor model of liver cancer (H22 cells). The developed CDs system is internalized into cancer cells and achieved targeted bioimaging in vivo. It selectively killed cancer cells and inhibited tumor growth remarkably without causing any obvious organ toxicity [128].

**Figure 5:**
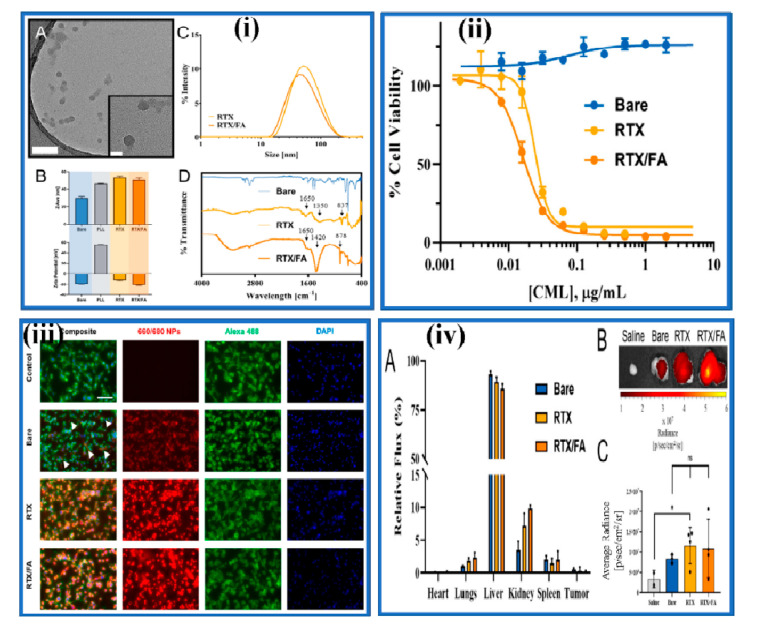
Illustration depicts the formulation design, in vitro and in vivo evaluation of folate-modified raltitrexed-loaded multifunctional nanoparticles (RTX/FA NP) as cancer theranostics. (**i**) Physical characterization of raltitrexed (RTX) and RTX/FA NP formulations; [A] TEM images, [B] mean size and zeta-potential, [C] intensity-derived size distribution, [D] IR spectra. (**ii**) Cell viability assay of CT26 cancer cells after 72 h treatment. (**iii**) In vitro cellular uptake of developed nanomedicine through fluorescence microscopy. (**iv**) Biodistribution of NP formulations injected into mice bearing CT26 tumors; [A] comparative profile of mean relative flux in various organ from different treatment group, [B] comparative profile of signal intensity of tumors from different treatment group, [C] comparative profile of mean average radiance of tumors from the different treatment group. Reproduced from [129], MDPI, 2020. * significant compared to control.

More recently, the researchers are also exploring the potential of different forms of organic nanomedicine such as polymeric nanoparticles, polymeric micelles, and liposomes, etc., for cancer-targeted delivery of nanotheranostics. Rosch et al. developed folate-modified raltitrexed-loaded multifunctional polymeric nanoparticles (RTX/FA NP) as theranostic nanomedicine against colorectal cancer (CRC) [129]. This investigation involves the formulation design, physicochemical characterization, cell viability assay, cellular uptake study, and in vivo biodistribution profile of the developed nanotheranostic system. The fluorescence microscopy and flow cytometry analysis indicated an enhanced accumulation of developed RTX/FA NP nanotheranostics in cancer cells (CT26 murine cells) and improved accumulation in CRC tumors (Illustrated in Figure 5).

#### 2.3.5. Revenue and Market Potential of Cancer Nanomedicines

Concerning the revenue and market potentiality of theranostics nanomedicines, as the absolute cancer theranostics have not been in advance clinical phases and have not been approved by regulatory agencies, the current status can only be assessed in terms of the revenue generated by the currently marketed cancer nanomedicines. Here, we give a glimpse of the market potential of the cancer nanomedicines currently prescribed in cancer therapy. Moreover, investments in an innovator company and to a specific nanomedicines product portfolio are the other indicators of the market perspective, which are also discussed.

The global nanomedicine market is anticipated to reach $350.8 B by 2025, according to a new report by Grand View Research. A review of the revenue generated by marketed cancer nanomedicines showed that the sales of the top approved nanomedicine products were brought in $950 M (Abraxane^®^; paclitaxel protein-bound nanoparticles injection suspension), $275 M (Depocyte^®^; Cytarabine liposome injection), $252 M (Doxil^®^/Caelyx^®^; doxorubicin-loaded PEGylated liposomes), and $122 M (ONIVYD^®^; irinotecan PEGylated liposomal formulation) in 2018. A recently approved cancer nanomedicine, Vyxeos^®^ (also known as CPX-351 in clinical phases; (only in US market-FDA granted regular approval in 2017) was brought in $75 M of the revenue in 1st year (2017–2018) itself after its launch. Vyxeos is a dual drug (daunorubicin and cytarabine) liposome use for the treatment of acute myeloid leukemia or AML. In the case of Abraxane, a 15% increase in net sales was recorded once compared to the year 2017. A nearly similar trend has been observed for the other approved products. In the case of Vyxeos, the forecast is almost double the 1st year revenue [135]. Investments in small or start-up firms dedicated to the area of cancer nanomedicine development is another key indicator of future market potential. However, it is out of the scope of this review (and it is difficult to track all the investments down the line of the company establishment), and we summarized here the noteworthy investments from venture capital (VC), capital markets, and large pharmaceutical corporations in this area. In this context, many capital-efficient companies developed cancer nanomedicine for orphan indications. One good example is Celator Pharmaceuticals who raised ~$170 M from VC, capital markets, and a grant from the Leukemia & Lymphoma Society for the development of products based on their proprietary CombiPlex technology platform [that came as approval of Vyxeos (discussed above) after phase III trial). Likewise, Oasmia Pharmaceutical raised ~$248.4 M for the development of Apealea (later in the year 2018 were approved by EMA for ovarian cancer). Alongside, it is significant to note that targeted cancer nanomedicines also attracted a sizable amount of investments and further interest of big innovator pharmaceuticals. For example, BIND Therapeutics (est. 2007), developing Accurins platform, raised $70.5 M from VC, $102.2 M from capital markets, and $705 M in large Pharma partnerships. However, later the pharma partner in the development of Amgen ended the contract because of the failure in trial phases. Merrimack (est. 2000) worked on the liposomal platform technology and raised $223 M from VC, $165 M from capital markets, and $1.643 B from partnerships [135]. It is important to note here that the new molecules (biologicals and small molecules drugs) are not the only focus areas where nanoparticles-based carriers are under development, other important driving forces lead to (and further expected to) increase the investments in the nanomedicines and thus potentially increasing the prospects in its clinical transition. In the current situation, pharmaceutical companies are facing financial pressures because of the expiry or end of their patents on major blockbuster innovator drug (NDA) and competition for their generics (and also nanosimilars or NBCPs). The expiration of patents is expected to cause a substantial drop in the revenue and product-associated investments for the innovators in the coming years. Furthermore, new drug development as replacements are not on the horizon in the very near future and any NCE itself has a very limited success rate [136]. Therefore, pharmaceutical companies may be forced to look at nanopharmaceuticals applications as a way to reformulate or develop new nano-based products for the failed and expiry or near to ending patents cover traditional drugs to create novel agents with favorable physicochemical, pharmacokinetics, or site specificities.

## 3. Challenges in Clinical Translation and Approval of Cancer Nanomedicine

It takes exceptionally extensive time and efforts to bring an anticancer nanomedicine from laboratory settings to clinics and its commercialization. There are fewer examples of the approved nanomedicines such as *Doxil^®^*, *Myocet^®^*, *Abraxane^®^*, *Depocyt^®^*, and *Genexol^®^* for chemotherapeutics which highlight the enormous gaps in the process of nanomedicine translation from laboratory to clinical and large-scale production at the industrial level [137].

The present discussion brings forth critical issues that are hampering the clinical development and market approval of anticancer nanomedicines. First, and foremost the major lacunae lie in our lack of profound knowledge of the pathophysiological complexities and heterogeneity of tumor sites that affect patient selection. Those patients are not even identified who are likely to benefit most from given nanomedicine-based chemotherapy [138]. Then next, the in vivo NPs behavior knowledge is restricted to animal data and the animal models used do not mimic the actual in vivo conditions. Usually, the NPs targeted for solid tumor after systemic administration, are accumulated in the tumor through the EPR effect (leaky tumor vasculature and poor lymphatic drainage). Nevertheless, several crucial aspects related to EPR interpretation have been greatly overlooked such as the influence of NP–protein interaction, blood circulation, tumor tissue penetration, and cellular internalization. Furthermore, all these biological processes are greatly affected by NPs properties (for example, size, geometry, surface features) thus there are so many factors governing the EPR effects-driven in vivo NPs behavior which cannot be predicted from animal data for humans. To date, there is not a single model that can completely replicate the entire facets of human malignancy [139,140,141]. Taking into consideration the phenomenon of tumor metastases on the way to cancer mortality, models of human tumor metastasis will be of substantial importance for the assessment of EPR and NPs penetration. The clinical translation of anticancer nanomedicines could see a breakthrough outcome via the introduction of animal models that can intently mimic the heterogeneity and anatomical histology of human tumors [142]. As we know the significance of optimized physicochemical parameters for the successful development of therapeutic NPs, here comes another big hurdle of speedy and reproducible synthesis of NPs in large quantities with distinctive characteristics. The large-scale and reproducible synthesis of complex NPs formulation is incredibly challenging as this includes numerous steps or intricate technologies. Therefore, for translation of NPs from laboratory to clinic requires a novel scale-up strategy with modifications in the optimization of physicochemical characterization parameters or alterations in formulation development method [143]. Another obstacle in clinical development is the obscurity in the chemistry, manufacturing, and controls (CMC) and good manufacturing practice (GMP) requirements as the scale-up of more complex nanomedicines may stance additional CMC and GMP compliance, and necessitate improvisation of prevailing unit operations [144].

In a nutshell, the translation of anticancer nanomedicine from laboratories to clinics and market approval could be made faster and smoother by addressing the highlighted issues of implicating clinically relevant animal models, careful and tumor-specific patient selection, the introduction of innovative technology for determination of physicochemical characteristics of NPs in large proportions.

### Toxicity Apprehension

In this review, we focused on the extensively studied inorganic (also includes metallic) NPs as cancer therapeutics, diagnostics, and theranostics. In absolute terms, it is the inorganic and metallic NPs owning to their unique physicochemical properties that can be called diagnostics and theranostics nanomedicines. Preclinical studies over the last two decade demonstrated that the inorganic NPs offer unique diagnostic and theranostics opportunities (that polymeric and lipidic NPs cannot offer) and poised to tackle many challenges which remained unaddressed in clinical settings. On the other hand, when looking in the context of safety point of view, toxicity concerns overshadowing their therapeutics and theranostics benefits. In general, nanomaterials tend to accumulate within various types of cells, including macrophage-type cells (both histiocytes and blood phagocytic cells) and RES cells in the body. Their deposition in tissues such as lymph nodes, bone marrow, brain, spleen, adrenals, liver, and kidneys, etc., are well established. Influence of the physicochemical properties (for example—size, shape, solubility, surface charge, chemical structure and reactivity, and surface modification) of inorganic NPs have extensive studies and are well-known [145,146]. A common safety issue with this category of nanomedicines has been known to induce DNA damage and oxidative damage [147,148,149,150]. We are discussing here the biological safety issues with commonly used (and considered as promising) inorganic NPs. First take the example of AuNPs; colloidal AuNPs with a particle size of 10–50 nm may cause greater toxicity than the larger size particles of 100–200 nm. Likewise, it was found that AuNPs with size ranging from 2.8 to 38 nm are more toxic and can induce immunological reactions [151]. However, AuNPs with a particle size of 15 nm was found to be nontoxic to cells, even at 60-fold higher concentrations than the IC50 of the smaller AuNPs [152]. These findings seemed to validate the size-dependent toxicity of AuNPs. Indeed, AuNPs, especially those of smaller sizes, significantly upregulated the expressions of pro-inflammatory genes, interleukin-1, interleukin-6, as well as tumor necrosis factor-alpha, which leads to a decrease in the population of macrophages [153]. Concerning the shape and its correlation to the toxicity of gold NPs, the findings published by Sun et al. demonstrated the effect of the shape of AuNPs on the in vivo toxicity [154]. It was found that the rod-shaped AuNPs are the most toxic, followed by cube-shaped AuNPs. The sphere-shaped AuNPs exhibited the best biocompatibility properties. Besides, the study revealed preferential accumulation of all AuNPs in the liver and spleen. It was furthermore found that positively charged spherical AuNPs demonstrated a more toxic effect than negatively charged particles of the same size. Like other nanomedicine’s biodistribution, AuNPs can be highly distributed in blood, brain, lungs, heart, kidney, liver, and spleen after crossing the small intestine by persorption mechanism [155,156,157]. Other popular inorganic NPs that have been extensively explored as theranostics are QDs. They are also reported with serious toxicity issues. Lung and kidney damage are known to be caused by QDs composed of heavy metal ions like Cd^2+^. The QDs without polymer protection shows the release of toxic cadmium on exposure to ultraviolet radiation [158,159,160,161]. In vitro toxicity studies of CdSe QDs on primary hepatocytes showed acute toxicity profile due to the liberation of free Cd2+ ions [162,163,164]. SPIONs are thought to be less toxic under in vivo conditions as they are considered to be biodegradable [165,166]. This is one of the most evaluated and evolved metallic nanomedicines and has been approved as a diagnostics application. Recently, magnetic hyperthermia outcome is an important breakthrough that further positioned its role as true theranostics. However, many iron oxide materials have been withdrawn due to toxicity concerns or lack of clinical benefit. The major issue remained the same as that been reported in the case of other metallic or inorganic NPs. Researchers have been in disagreement regarding the level of toxicity mainly based on viability test results. The biodistribution of SPIONs showed its distribution to different tissues and organs, including the brain; nevertheless, acute toxicity, genotoxicity, immunotoxicity, reproductive, and neurotoxicity do not provide a clear view and have discrepancies in results in different animal models. Lastly, it is important to discuss gadolinium, a common contrast agent use in clinical settings including in cancer therapy. Interestingly, gadolinium NPs have been proposed as theranostics as well and showed promising results in pre-clinical in-vivo. However, it is now not only reported to be associated with human toxicity. The patients associated with pre-existing kidney failure may end up having systemic tissue fibrosis and lead to nephrogenic systemic fibrosis (NSF) due to gadolinium application.

## 4. Expert Opinion and Future Directions

**Figure 6:**
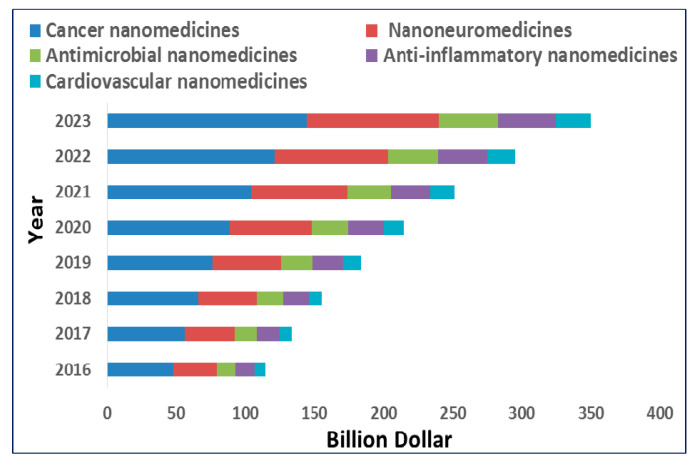
Illustration depicts worldwide nanomedicine market revenue in terms of different therapeutics areas (source; based on market data forecast and Infoholics Report December 2017).

In this opinion section, we are going to cover two main areas of cancer nanomedicines; cancer nanomedicines therapeutics and the nanotheranostics. Continuous improvements in cancer treatment have been made over the past four decades with the development of cancer nanomedicines, including prolonged overall survival, increased quality of life, and reduced toxicity. Since the approval of the first cancer nanomedicine Doxil^®^ two decades ago, a huge investment and research works have been carried out at the industrial and academic research level toward the development of cancer nanomedicines. One can argue and indeed it is challenging to convince that the cancer nanomedicines fulfilled the high expectations in terms of clinical success compared to what has been presented in pre-clinical outcomes. Clinical trial failures resulted in product terminations and business insolvency. Yet, recent approvals of nanomedicine products for orphan cancers and the continuing development of cancer nanomedicines for cancer immunotherapy remained a high motivation for academic and industrial research in this area. In late December 2020 approval (in USA, EU, UK, and many other parts of the work) of mRNA carrying liposomes as a vaccine for COVID-19 fueled the continuing industrial and academic interest in cancer nanomedicines and renewed their hope in the path of development of non-viral cancer vaccine. Since the time of its development, nanomedicines have attracted significantly more interest in the cancer therapeutic area compared to any other area of therapy. The Global Nanomedicine Market Revenue trends since 2016 and the forecast further indicate that in the general the market is growing for nanomedicine and it has a significantly higher share of cancer nanomedicines and is way ahead of other therapeutic areas (illustrated in Figure 6).

With 18 approved cancer nanomedicines and many with the positive outcome in phase III, it should be clear that cancer nanomedicines will continue to improve cancer treatment. Furthermore, the successful approval of many nano-similars of cancer nanomedicines is reducing the high financial burden of the cancer treatment, and it is very hopeful that in near future much more generic version will be available to improve this situation further and will make nanomedicine more affordable.

Nanotheranostic field is a growing research field that is yet to meet the clinical standards. For example, some of the nanotheranostic systems though demonstrate significant diagnostic efficacy but lack therapeutic competence. Other systems have demonstrated the main therapy index with low imaging competence. However, the researcher has shown great efforts to translate these new systems into clinical trials by investigating different nanomaterials or modification techniques and assessing their in vivo performance. Most of the present investigations assess the diagnostic and therapeutic application of nanotheranostics exploiting in vivo animal models, demonstrating promising results. However, if this system is administered to a human subject, their application generally fails because of the difference in the diffusion mechanism of nanotheranostic in the case of animal and human models. Another issue related to this system is their possible toxicity and safety concern in case of use in humans. The CNTs and metallic NPs-based nanotheranostic system raised the concern of safety because of their slow degradation and in vivo fate. Consequently, various attempts have been utilized to surface coating this system with biocompatible/biodegradable polymer or synthesis of nanotheranostic system utilizing the clinically approved nanomaterials to improve its in vivo efficacy in a human subject. Compared to the past, many sophisticated nanotheranostic systems have been designed worldwide. Indeed, massive progression should be carried out before their clinical application. To conclude, although a great effort is still needed, the future of nanotheranostics utility in clinical practice is near.

## 5. Conclusions

Cancer nanotechnology has evolved significantly over the years and provided us a new class of medicine in cancer chemotherapy termed “nanomedicines” which are considered safer, effective, and has better patient amenability. While considering the theranostic applications in cancer therapy, metallic and carbon-based nanoparticles have been comprehensively investigated and showed noteworthy outcomes in pre-clinical studies. Substantial research activity, increased number of approval for clinical use from regulatory authorities, etc., eventually raise their long-term socio-environmental impact and toxicity concern that needs to be addressed. Manufacturing process, processing add, residual chemicals from the formulations are some of the key attributes (together with the physicochemical properties of the metallic and other in-organic nanoparticles) may have the potential to induce toxicity at cellular and sub-cellular level, and may also cause physiological and metabolic alteration. The correlation and reproducibility between the toxicity testing methods used in nanomedicine are still poorly presented. Challenges in toxicity assessment present inadequate in vivo findings and poor in vitro, in vivo correlation because of the lack of appropriate tools to directly interrogate nanomedicine in a complex biological system. As it is necessary to transform and update the required bio-pharmaceutical and toxicity regulation, the regulatory agencies are moving rapidly forward to the new metrics to keep pace with the changing paradigms introduced by nanomedicines.

## Figures and Tables

**Table 1:** Some of the cancer nanomedicines (approved or in clinical trial stages), their characteristics, and indications.

Cancer Nanomedicines	Features	Indications (Approved and or in Clinical Phases)
DaunoXome^®^	Daunorubicin citrate encapsulated liposomes of size less than 100 nm/EPR-based passive targeting to tumor	HIV-associated Kaposi’s sarcoma
DepoCyt^®^	Cytarabine encapsulated in multivesicular liposomes Sustained release formulation of cytarabine maintains cytotoxic concentrations of the drug in the cerebrospinal fluid for more than 14 days after a single 50 mg injection	lymphomatous malignant meningitis
Doxil^®^	Doxorubicin hydrochloride encapsulated in stealth liposomes (~100 nm)/passive targeting to tumor via EPR effect	AIDS-associated Kaposi’s sarcoma, multiple myeloma, ovarian cancer
Marqibo^®^	Vincristine sulfate encapsulated in liposomes (~100 nm)/passive targeting to tumor via EPR effect	Acute lymphoid leukemia
Mepact™	Mifamurtide incorporated into large multilamellar liposomes/mononuclear phagocytic system (MSP) targeting	Non-metastasizing osteosarcoma
Myocet^®^	Doxorubicin encapsulated ~180 nm oligolamellar liposomes/MPS targeting; “MPS depot,” slow release of drug into blood circulation resembles	Metastatic breast cancer
Oncoprex (Genprex)	Tumor suppressor gene *TUSC2*/*FUS1* encapsulated liposome (~100 nm)/passive targeting to tumor via EPR effect	lung cancer/*Clinical phase I/II*
Oncaspar^®^	It is a pegaspargase; a PEGylated form of asparaginase for i.m injection	Acute lymphoblastic leukemia
Eligard^®^	PLGA-based leuprolide acetate (synthetic GnRH) microparticles suspension for s.c injection.	Advanced prostate cancer
Genexol^®^	Paclitaxel loaded PEG-PLA block copolymer-based micelles (20–50 nm)/passive targeting to tumor via EPR effect	Metastatic breast cancer, pancreatic cancer
Opaxio^®^	Paclitaxel covalently linked to polyglutamate-based solid NPs/passive targeting to tumor via EPR effect and drug release inside solid tumor via enzymatic hydrolysis of polyglutamate	Glioblastoma
Halaven E7389-LF^®^	Eribulin mesylate liposomal formulation/passive targeting via EPR	Solid tumors/*Clinical phase I*
Abraxane^®^	Paclitaxel-loaded albumin NPs (<150 nm)/passive targeting via EPR and dissociation of drug-albumin bound helps endothelial transcytosis of paclitaxel via albumin-receptor	Metastatic breast cancer, non-small-cell lung cancer
NanoTherm^®^	Aminosilane-coated superparamagnetic iron oxide NPs (~15 nm)/passive targeting to tumor via EPR, the injected NPs oscillate, generating heat directly within the tumor tissue once exposed to the alternating magnetic field	*Clinical phase IV* approval (EU) for local heat ablation in glioblastoma, prostate, and pancreatic cancer
ThermoDox^®^	Doxorubicin lysolipid heat-sensitive liposome (~100 nm)/passive targeting to tumor via EPR on i.v injection; when heated to 40 °C–45 °C, liposome releases doxorubicin directly into and around the targeted tumor	Breast cancer, primary liver cancer/*Clinical phase III*
Feridex, Resovist, Combidex (*withdrawn*), Clariscan (*withdrawn*) and Feraheme	Iron oxide NPs/passive targeting to tumor via EPR/Feridex^®^ cannot be administered as an i.v bolus; severe back pain, while Resovist^®^ can be administered by fast bolus injection, and therefore imaging of the arterial phase is feasible. Feraheme (Ferumoxytol) iron oxide NPs has a carbohydrate coat. The agent is taken up by macrophages and ultimately the RES	MRI imaging of lymph nodes and certain liver tumor
Lipocurc	Liposomal curcumin of size <100 nm/passive targeting by EPR	Solid tumors/*Clinical phase I/II*
MM-302	HER2-targeted PEGylated liposomal doxorubicin/active and passive targeting to the tumor.	Breast cancer/*Clinical phase II/III*
AuroLase^®^	PEG-coated silica-gold nanoshells for near IR thermal ablation	Thermal ablation of solid tumors & metastatic lung tumors/*Phase I*
ABI-009	Rapamycin loaded albumin NPs/passive targeting and endothelial transcytosis via albumin-receptor	Bladder cancer/*Clinical phase I/II*
CRLX301	Cyclodextrin-based nanoparticle- docetaxel conjugate	Solid tumors/*Clinical phase I/II*
NC-6004 Nanoplatin	Polyamino acid and cisplatin derived PEGylated nano micelles	Solid tumors, lung, bladder, or pancreatic cancers/*Clinical phase II/III*
AZD2811	Aurora B kinase inhibitor loaded long-circulating NPs	Solid tumors/*Clinical phase I*
Promitil	Mitomycin-C loaded PEGylated liposomal formulation	Solid tumors/*Clinical phase I*

**Table 2:** Summary of recent nanomedicine products for therapeutic, diagnostic, and theranostic applications in cancer and their in vitro/in vivo outcome.

S.No.	Type of Cancer Nanomedicine and Overview	Purpose in Cancer Management	In Vitro/In Vivo Outcome	Ref.
1.	Hypericin-bearing magnetic iron oxide nanoparticles	Therapeutics	Upon irradiation, light-induced cell death showed concentration and time-dependent death of Jurkat T cells because of the generation of reactive oxygen species	[22]
2.	Thiolated heparin–pheophorbide A (PhA) conjugated magnetic iron oxide/gold hybrid nanoparticle	Therapeutics	Phototoxicity and strong fluorescence signals from the NPs result in A549 cells deaths under light irradiation.	[23]
3.	Anti-HER2 (c-erbB-2) conjugated gold nanoshell	Therapeutics	Nanocomplexes when targeted to OVCAR3 cells and irradiated with near infra-red (NIR) laser-caused selective destruction of cancer cells.	[39]
4.	Palladium-gold nanostructures	Therapeutics	-Excellent in vitro and in vivo anticancer activity in HeLa cells and HeLa tumor xenograft of male B9 mice at laser radiations of 808 nm	[41]
5.	Magnetic nanoparticle clusters	Therapeutics	-Excellent in vitro cytotoxicity in A549 cells as well as in A549 tumor-bearing mice model upon NIR irradiation at 808 nm	[42]
6.	Platinum (II) drug-loaded gold nanoshells	Therapeutics	-Upon NIR irradiation, gold-nanoshells promote a significant increment in temperature that was found sufficient to ablate the tumor cells. -Platinum (II) drug-loaded gold nanoshells exhibited a profound inhibition of tumor growth compared to chemotherapy or photothermal therapy given alone.	[130]
7.	PDPN antibody and doxorubicin (DOX) conjugated gold nanoparticles [(PDPNAb)-AuNP-DOX]	Therapeutics	-(PDPNAb)-AuNP-DOXshowed good biocompatibility, drug loading capacity, cellular uptake efficiency, a pH-dependent drug release profile, and a much lower IC_50_ than free DOX. -The designed (PDPN Ab)-AuNP-DOX system can be applied as a PTT platform because of its high photothermal conversion efficiency. -The administration of (PDPN Ab)-AuNPDOX followed by laser irradiation exhibited an enhanced antitumor effect in in vitro and in vivo model.	[131]
8.	Cetuximab decorated doxorubicin encapsulated magnetic graphene oxide nanoparticles	Therapeutics	-Improved cellular uptake in CT-26 cells via EGFR receptor-mediated endocytosis. -Improved photothermal ablation upon NIR irradiation. -Significant improvement in tumor volume reduction in CT-26 tumor-bearing BALB/c mice.	[132]
9.	Folic acid-modified PEGylated polyethyleneimine (PEI)-entrapped gold nanoparticles (FA-Au PENPs)	Diagnostics	-FA-Au PENPs showed the excellent potential to target FA receptor overexpressed KB-HFAR and KB-LFAR cancer cells -Significantly enhanced in vitro and in vivo CT contrast enhancement compared to non-targeted Au PENPs	[61]
10.	Lactobionic acid (LA)-modified dendrimer-entrapped gold nanoparticles (LA-Au DENPs)	Diagnostics	-LA-Au DENPs showed significantly higher cellular internalization in asialoglycoprotein receptors HepG2 and L929 cancer cells -Significantly enhanced in vitro and in vivo CT contrast enhancement compared to non-targeted Au DENPs	[62]
11.	Arginine-glycine-aspartic acid (RGD) peptide-targeted iron oxide nanoparticles	Diagnostics	-Significantly higher in vitro and in vivo r2 relaxivity and selective targeting to the α_v_β_3_ receptor overexpressed U87MG cancer cells	[73]
12.	Polyethyleneimine (PEI)-stabilized hyaluronic acid (HA)-tagged magnetic iron oxide nanoparticles	Diagnostics	-Specifically internalized in CD44 receptor-overexpressed Hela cells -Significantly higher r2 relaxivity and contrast in T2-weighed MR imaging in a tumor model	[74]
13.	Tumor-avid cyclic pentapeptide labeled (Arg-Gly-Asp-DPhe-Lys) NIR emitting silver sulfide quantum dots (QDs)	Diagnostics	-Fluorescence microscopy revealed selective integrin-mediated internalization of targeted quantum dots in 4T1luc cancer cells	[78]
14.	BRCAA1 antibody- and Her2 antibody-conjugated amphiphilic polymer engineered CdSe/ZnS QDs	Diagnostics	-The developed QDs exhibited strong photoluminescence and revealed targeted imaging of in vivo gastric cancer (MGC803) cells	[79]
15.	Pullulan-based gadolinium-chelated nanoparticles	Diagnostics	-Gadolinium nanoparticles showed early detection of the tumor (~3 mm^3^) within 2 h after the administration of nanoparticles within the small CT26 tumor-bearing mice model by MRI and optical imaging.	[80]
16.	Gold-coated iron oxide nanoparticles (GIONPs)	Diagnostics	-The developed GIONPs showed reduced cytotoxicity, produce a negative T_2_ signal in the MRI, which makes them a suitable candidate as a contrast agent for MRI applications, and validated in small animals.	[133]
17.	^99 m^Tc-gallic-gold nanoparticles	Diagnostics	-^99 m^Tc-gallic-gold nanoparticles displayed good stability and cytocompatibility. -It exhibited high uptake in tumor cells after intratumoral and intravenous injection.	[11]
18.	hyaluronic acid (HA)-functionalized GdCl_3_ entrapped SWNTs to deliver the contrast agent,	Diagnostics	GdCl_3_-bearing HA-SWNTs showed significantly higher circulation time for MRI. In vivo imaging in S180 cells, tumor-bearing male BALB/c mice revealed that Gd/HA-SWCNTs exhibited the highest tumor-targeting efficiency and T1-relaxivity enhancement.	[92]
19.	Epirubicin-5TR1 aptamer-SPIONs tertiary complex	Theranostics	-Significantly enhanced cellular uptake and cytotoxicity against C26 cells. - MRI demonstrated a high accumulation of the nano-magnets within the tumor site	[103]
20.	pheoA (photosensitizer) conjugated heparin-iron oxide nanoparticles (PheoA–Hep–Fe3O4 NPs)	Theranostics	- Significantly higher T2 signal intensity and cellular uptake of the NPs in KB cells -Excellent dose and time-dependent cytotoxicity	[104]
21.	Folic acid conjugated Protoporphyrin IX (photosensitizer) linked synthesized a near-infrared (NIR) emitting gold quantum cluster capped with lipoic acid	Theranostics	-Photodynamic therapy revealed a higher generation of singlet oxygen generation and the better localization of the NPs on tumor cells. -In vivo study with C6 cell line xenograft of Albino Swiss mice showed that the developed nanocluster is useful for the effective destruction and monitoring of tumor cells.	[112]
22.	Tungsten sulfide (WS2) quantum dots (QDs)	Theranostics	-The developed tungsten sulfide-quantum dots demonstrated a significant increase in signal intensity of X-ray computed tomography/photoacoustic imaging. -The developed system exhibited a synergistic effect of remarkable photothermal therapy/radiotherapy against tumor cell (in HeLa and HepG2 cells).	[115]
23.	Multi-walled carbon nanotubes (MWNTs) coated with manganese oxide (MnO) and polyethylene glycol (PEG)	Theranostics	-Upon NIR laser irradiation multi-walled carbon nanotubes coated with manganese oxide and polyethylene glycol exhibited high photothermal conversion efficiency. -The developed system exhibited powerful dual-modality for lymphatic tracing capability and high efficiency for tumor ablation in in vivo tumor model.	[124]
24.	PEGylated graphene oxide-BaGdF_5_ nanocomposites (GO/BaGdF_5_/PEG)	Theranostics	-The developed nanotheranostic system demonstrated a positive magnetic resonance contrast effect and improved X-ray attenuation characteristics than Iohexol. -It enables effective dual-modality for MRI and X-ray computed tomography imaging against in vivo tumor models.	[125]
25.	^99 m^Tc-doxorubicin-loaded gallic acid-gold nanoparticles (^99 m^Tc-DOX-GA-Au-NPs)	Theranostics	-GA-Au-NPs exhibited increased anti-proliferative activity, with approximately a four-fold lower IC_50_ value compared to free DOX. The optimized radiolabeling efficiency of ^99 m^Tc-DOX was ≈93%. It showed good physiological stability in mice serum for at least 8 h. -The intratumoral delivery of ^99 m^Tc-DOX-GA-Au-NPs in the tumor-induced mice model showed dramatic improvement in the accumulation of the drug in the tumor.	[12]
26.	LDH-stabilized hyaluronic acid-modified ultrasmall iron oxide nanoparticles loaded with doxorubicin	Theranostics	-The LDH-Fe_3_O_4_-HA nanohybrids possess good colloidal stability and cytocompatibility, display an r_1_ relaxivity ten-fold higher than the pristine ultrasmall Fe_3_O_4_. -In vitro experiments demonstrated that LDH-Fe_3_O_4_-HA/DOX nanohybrids can specifically target B16 cells overexpressing CD44 receptors and effectively release DOX to the nucleus. -In vivo results show that with the pretreatment of tumor tissue by HAase to degrade the overexpressed HA in the extra-cellular matrix, the designed nanoplatforms have a better tumor penetration for significantly enhanced MR imaging ability of tumors and improved therapeutic outcome with low side effects.	[134]
